# Gene flow in the green mirid, *Creontiades dilutus* (Hemiptera: Miridae), across arid and agricultural environments with different host plant species

**DOI:** 10.1002/ece3.510

**Published:** 2013-02-25

**Authors:** J P Hereward, G H Walter, P J DeBarro, A J Lowe, C Riginos

**Affiliations:** 1 School of Biological Sciences, The University of QueenslandBrisbane, Qld, 4072, Australia; 2 CSIRO Ecosystem SciencesGPO Box 2583, Brisbane, Qld, 4001, Australia; 3 Cotton Catchment Communities CRC, Australian Cotton Research InstituteLocked Mail Bag 1001, Narrabri, NSW, 2390, Australia; 4 Australian Centre for Evolutionary Biology and Biodiversity, School of Earth and Environmental Sciences, University of AdelaideSA, 5005, Australia; 5 State Herbarium of South Australia and Science Resource Centre, Department of Environment and Natural ResourcesHackney Road, SA, 5005, Australia

**Keywords:** Agriculture, dispersal, gene flow, host plant, human-altered landscapes, insect herbivore, microsatellite, migration, mitochondrial

## Abstract

*Creontiades dilutus* (Stål), the green mirid, is a polyphagous herbivorous insect endemic to Australia. Although common in the arid interior of Australia and found on several native host plants that are spatially and temporally ephemeral, green mirids also reach pest levels on several crops in eastern Australia. These host-associated dynamics, distributed across a large geographic area, raise questions as to whether (1) seasonal fluctuations in population size result in genetic bottlenecks and drift, (2) arid and agricultural populations are genetically isolated, and (3) the use of different host plants results in genetic differentiation. We sequenced a mitochondrial COI fragment from individuals collected over 24 years and screened microsatellite variation from 32 populations across two seasons. The predominance of a single COI haplotype and negative Tajima D in samples from 2006/2007 fit with a population expansion model. In the older collections (1983 and 1993), a different haplotype is most prevalent, consistent with successive population contractions and expansions. Microsatellite data indicates recent migration between inland sites and coastal crops and admixture in several populations. Altogether, the data suggest that long-distance dispersal occurs between arid and agricultural regions, and this, together with fluctuations in population size, leads to temporally dynamic patterns of genetic differentiation. Host-associated differentiation is evident between mirids sampled from plants in the genus *Cullen* (Fabaceae), the primary host, and alternative host plant species growing nearby in arid regions. Our results highlight the importance of jointly assessing natural and agricultural environments in understanding the ecology of pest insects.

## Introduction

Many insects that damage agricultural crops have invaded the resources provided by agriculture across wide areas and this has generated alternative predictions as to their evolutionary trajectories. Although the provision of novel resources by agriculture might promote host adaptation (Via 1990), it has also been argued that gene flow will increase among populations of native insects when their range is expanded through the anthropogenic spread of potential hosts, making local adaptation less likely (Oliver 2006). Insects that use native and introduced hosts thus provide a “natural experiment” to explore the likely consequences of ongoing anthropogenic change in plant distribution and abundance.

Few genetic studies have examined the interactions of insects between native host plants and agricultural resources simultaneously, but the available evidence indicates that several outcomes are possible, including geographic differentiation, host-associated differentiation, and widespread gene flow. Both the rice mirid *Stenotus rubrovittatus* (Hemiptera: Miridae), native to Japan, and Queensland fruit fly *Bactrocera tryoni* (Diptera: Tephritidae) show strong geographic differentiation (Yu et al. 2001; Kobayashi et al. 2011). In the former, it indicates divergence across Pleistocene refuges and the latter divergence as invading crop and fruit hosts outside its original Queensland distribution. Furthermore, an isolated fruit fly population in inland Australia (Alice Springs) showed strong genetic evidence of a population bottleneck. Host-associated differentiation has also been recorded in the corn leafhopper *Dalbulus maidis* (Hemiptera: Cicadellidae), and this has been associated with a shift from wild hosts to maize (*Zea mays*) within the last 9000 years since domestication (Medina et al. 2012). In contrast to the above examples, a lack of isolation by distance was found across 1700 km in the migratory moth *Trichoplusia ni* (Lepidoptera: Noctuidae), between its native range in California, and crops that it seasonally invades in Canada (Franklin et al. 2010). Clearly, the patterns found to date are strongly influenced by the biology and life history of the organism in question, as well as the environment it inhabits.

Strong regional differentiation, as found in *Stenotus rubrovittatus* (Kobayashi et al. 2011), and Queensland fruit fly (Yu et al. 2001), might be expected in species that do not regularly disperse long distances (Bohonak 1999). Conversely, high gene flow, as documented for the migratory moth *Trichoplusia ni* (Franklin et al. 2010), has also been reported in many widespread agricultural pests (Endersby et al. 2006, 2007; Margaritopoulos et al. 2009), and even in pest species thought to be relatively sedentary (Voudouris et al. 2012). Anthropogenic and unassisted dispersal can both allow the invasion of novel resources by insect populations (Stone and Sunnucks 1993; Stone et al. 2007). Such anthropogenic dispersal was thought to be the primary mechanism allowing colonization of grain storages by *Tribolium castaneum*, as this species was considered relatively sedentary (Drury et al. 2009). Active dispersal by flight has subsequently been shown to better explain patterns of regional genetic differentiation (Ridley et al. 2011; Semeao et al. 2012), highlighting that the capacity of organisms to disperse can be underestimated.

Another important aspect of pest insect dynamics is fluctuations in population size, which are expected based on the seasonal availability of most agricultural crops and the occurrence of pest outbreaks. Temporal fluctuations in gene frequencies are tied to the number of effective breeders (Waples and Teel 1990), and the temporal stability of regional genetic structure recorded across 5 years of sampling in the Queensland fruit fly implies that populations of sufficient size persist across seasons, despite the occurrence of regional outbreaks of this species (Yu et al. 2001). Regional differences in outbreak propensity in the migratory locust (*Locusta migratoria*) have allowed an empirical evaluation of its effects (Chapuis et al. 2008, 2009). No difference was found in genetic diversity between outbreak and non-outbreak populations (indicating that non-outbreak populations persist in sufficient size), but regional differentiation was much higher for non-outbreak populations (Chapuis et al. 2008, 2009). Spatial and temporal variance in population size, migration rates, and extinction rates are predicted to not only affect mean *F*_ST,_ but also result in large fluctuations in the genetic differentiation between populations over time (Whitlock 1992).

Patterns of host-associated differentiation might be obscured by migration, bottlenecks, and population expansion, and interpreting the relative effects of demographic processes remains a challenge for empirical population genetics (Pavlidis et al. 2008; Li et al. 2012). Furthermore, host plant-associated differentiation following host shifts is considered more likely in host specialists (Funk et al. 2002). Few studies report host-associated differentiation in insects that use multiple host plants (but see Sword et al. 2005). Geographic differentiation generally appears to be higher in host plant specialists than insects that use multiple hosts (Kelley et al. 2000; Zayed et al. 2005; Gaete-Eastman et al. 2007; Habel et al. 2009; Groot et al. 2011). This correlation is usually interpreted as a consequence of the spatial patchiness of a single resource in comparison to the more widespread availability of multiple resources. Resources can, however, be temporally patchy and it is not clear whether this correlation would hold under these circumstances.

In this study, we examine dispersal, fluctuations in population size, and the use of multiple host plants in *Creontiades dilutus,* the green mirid, across its native range (and host plants) in arid regions of Australia, and novel hosts (agricultural crops) that it has invaded within the last 200 years. We sampled mirids across both arid and agricultural regions in Australia, covering most of the geographic distribution of this endemic species and including the major host plants. We genotyped microsatellites from samples spanning two seasons and sequenced a mitochondrial COI fragment from green mirids collected over 24 years. Given the ecology of green mirids outlined below, and the challenges posed by both agricultural and arid environments, we structured our analyses according to the following three questions: (1) Do seasonal fluctuations in population size in both arid and agricultural regions result in genetic signatures of bottlenecks and drift? (2) Does long-distance dispersal occur between arid and agricultural populations? (3) Is genetic differentiation associated with the use of multiple host plants in the (arid) native range? We found that genetic patterns in *C. dilutus* are temporally dynamic, consistent with spatial and temporal heterogeneity in its arid range. Long-distance dispersal between arid and agricultural populations is evident from the data, and host-associated differentiation was found between the primary host plants and alternative hosts in arid regions. Together, these results highlight the importance of considering ecological and evolutionary processes across the distribution of an organism.

## Materials and Methods

### Study system

*Creontiades dilutus* is a mirid bug that is endemic to Australia and has become a major pest of several agricultural crops (Malipatil and Cassis 1997; McColl et al. 2011). This species has been associated with numerous host plant species and, prior to the advent of agriculture in Australia, was probably restricted to the relatively open interior. Here, the temporal variability of rainfall events is higher than in most other globally comparable desert systems with similar mean annual rainfall (Morton et al. 2011). Variability is compounded by years of drought (Nicholls 1991; Letnic and Dickman 2006). The availability of herbaceous plants is consequently ephemeral and often spatially patchy. During the summer months of November to February, *C. dilutus* persists in this region in low numbers despite temperatures in excess of 45°C and the availability of few host plants (JPH pers. obs., January 2007). Host plants persist longer in winter (June–August), but only if sufficient rain falls. With the exception of “flood years,” when host plants may be unusually widespread and persistent, suitable hosts generally require two or more successive rain events to thrive. These rain events usually occur only locally and typically the hosts are spatially patchy, with large areas of barren land between. Inland temperatures are close to optimal for mirid development during winter, allowing a generation time of around 25 days (Khan et al. 2009). Rapid population expansion is thus possible and large numbers of *C. dilutus* can be found where conditions are suitable.

*Creontiades dilutus* presumably relies primarily on dispersal to cope with adverse conditions by locating suitable patches of host plants when local conditions become unfavorable, because diapause (based on current evidence) is a facultative winter reproductive phenomenon (Miles 1995). Although *C. dilutus* persists throughout the year in the arid interior and sub-coastal agricultural regions of Australia, its abundance is seasonally inverse between the two, but the possibility of long-distance dispersal remains untested. In inland Australia, *C. dilutus* relies on host plants that are both spatially and temporally highly variable, even within a single season. Local population extinctions and founder effects might be expected, especially during dry years, as local resources die off and new patches are located. Conversely, when inland Australia experiences floods, the increased host abundance, together with the short generation time of this multivoltine insect, is likely to allow massive increases in population size across large areas. Pesticides applied in agricultural regions also have the potential to cause localized population contractions, and although green mirids are present on lucerne throughout the year in agricultural regions, their abundance during winter months is low (Miles 1995).

### Mitochondrial DNA sequencing and analysis

Ten population samples were taken during 2006 and 2007 from across Australia, and 146 of these individuals were sequenced for the COI fragment using the Folmer primers LCOI490 and HC02198 (Folmer et al. 1994) and a standard PCR protocol with an annealing temperature between 47°C and 50°C. We also obtained pinned specimens retained at The University of Queensland from previous research on this species. We were able to amplify the same fragment from 16 individuals collected in Gatton (Queensland) from lucerne in 1983 and 25 individuals collected in Biloela (Queensland) from lucerne in 1993. Details of the sample locations and Genbank accessions are provided in Table 1. DNA was extracted from the pinned specimens using DNeasy Blood and Tissue kit (Qiagen, Hilden, Germany) after being soaked in TE buffer overnight. The PCR protocol was the same as for the ethanol preserved specimens. The COI fragments were sequenced bi-directionally at Macrogen (Korea) on an ABI3730, and then aligned, edited, and trimmed using Codon Code Aligner v4.0 (CodonCode Corporation, Dedham, MA).

**Table 1:** Sampling locations for the 187 individuals for which the COI fragment was sequenced, including the number of sequences analyzed (*N*) and the Genbank accession numbers

Location	Date	Lat (S)	Long (E)	Host plant	*N*	Genbank Accessions
Adelaide	2/12/2006	−34.8208	138.8700	*Polygonum convolvulus*	8	JX186015 to JX186022
Balingup	14/09/2007	−33.7889	115.9760	*Solanum nigrum*	8	JX186023 to JX186030
BarcLong	16/08/2006	−23.5332	145.0765	*Cullen cinereum*	8	JX186031 to JX186038
Biloela	10/01/2007	−24.3739	150.5130	Gossypium hirsutum*	10	JX186039 to JX186048
Biloela	10/01/2007	−24.3739	150.5130	Medicago sativa*	8	JX186049 to JX186056
Emerald	14/08/2006	−23.4958	148.1884	*Verbesina enceliodes*	8	JX186057 to JX186064
Emerald	15/08/2006	−23.5722	148.1001	*Verbesina enceliodes*	4	JX186065 to JX186068
Emerald	15/08/2006	−23.4663	148.0918	*Vicia sativa*	8	JX186069 to JX186076
Kununurra	28/08/2006	−15.6459	128.6969	Gossypium hirsutum*	5	JX186077 to JX186081
Longreach	17/08/2006	−23.4177	144.2274	*Cullen cinereum*	8	JX186082 to JX186089
Longreach	17/08/2006	−23.4038	144.2212	*Cullen cinereum*	8	JX186090 to JX186097
Longreach	17/08/2006	−23.4382	144.2458	*Medicago polymorpha*	8	JX186098 to JX186105
Longreach	17/08/2006	−22.8941	143.7867	*Swainsona galegifolia*	8	JX186106 to JX186113
Narrabri	22/01/2007	−30.2008	149.5724	Gossypium hirsutum*	6	JX186114 to JX186119
Narrabri	22/01/2007	−30.2008	149.5724	Medicago sativa*	7	JX186120 to JX186126
Walget	31/08/2006	−29.9124	146.9179	*Rapistrum rugosum*	8	JX186127 to JX186134
WintJun	18/08/2006	−22.4120	143.0585	*Cullen cinereum*	7	JX186135 to JX186141
WintJun	19/08/2006	−23.7810	142.4658	*Cullen cinereum*	4	JX186142 to JX186145
WintJun	19/08/2006	−23.7338	142.4287	*Senna artemisioides*	5	JX186146 to JX186150
Biloela	20/09/1993	−24.3739	150.5130	Medicago sativa*	12	JX186151 to JX186162
Biloela	5/05/1993	−24.3739	150.5130	Medicago sativa*	13	JX186163 to JX186175
Gatton	16/01/1983	−27.5876	152.3618	Medicago sativa*	16	JX186176 to JX186191
Byee	14/03/2006	−26.2566	151.8539	*Cajanus cajan*	10	EF016724 to EF016733

* indicate samples from agricultural host plants.

Haplotype networks were constructed using the R package TempNet (Prost and Anderson 2012). The temporal haplotype network was restricted to sites in the eastern Queensland cropping region (48 samples from 2006/2007) where the samples from 1983 and 1993 had been collected. Nucleotide and haplotype diversity were calculated in DnaSP v. 5 (Librado and Rozas 2009).

### Microsatellites – sample collection and genotyping

A total of 32 population samples were collected from 17 different host plant species in inland Australia and sub-coastal eastern Australia between January 2007 and March 2008 (Table 2). Individual insects were preserved in 96% ethanol. DNA was extracted using a modified salt precipitation protocol based on that of Miller et al. (1988). Nine microsatellites (mirsat-2F, mirsat-4B, mirsat-3E, mirsat-A1, mirsat-3H, mirsat-6B, mirsat-5C, mirsat-G8, and mirsat-7G) were PCR amplified and genotyped on a Megabace capillary electrophoresis system (Amersham Biosciences) as per (Andris et al. 2010). Microsatellite peaks were confirmed and binned manually. In total, 768 specimens were genotyped; the DNA extractions of individuals that failed to amplify at more than six loci were assumed to be low quality and were discarded, leaving 665 genotyped individuals (Table 2), subsequent quantification of the samples with poor amplification revealed that the DNA was lower quality.

**Table 2:** Population codes, number of individuals genotyped, collection details, and host plant species for population samples used in this study

Code	*N*	Location	Date	Lat (S)	Long (E)	Host Plant	Family
BIL-GH*	19	Biloeala	9/01/2007	−24.3739	150.5130	*Gossypium hirsutum*	Malvaceae
BIL-MS1*	16	Biloeala	9/01/2007	−24.3739	150.5130	*Medicago sativa*	Fabaceae
BIL-MS2*	15	Biloeala	28/07/2007	−24.3770	150.5231	*Medicago sativa*	Fabaceae
EMR-VE1*	24	Emerald	10/01/2007	−23.5723	148.1002	*Verbesina encelioides*	Asteraceae
EMR-MS*	29	Emerald	10/01/2007	−23.5772	148.2084	*Medicago sativa*	Fabaceae
EMR-GH*	26	Emerald	10/01/2007	−23.4663	148.0916	*Gossypium hirsutum*	Malvaceae
EMR-CA*	21	Emerald	29/07/2007	−23.4657	148.0923	*Cicer arietinum*	Fabaceae
EMR-VE2*	26	Emerald	29/07/2007	−23.5558	148.1139	*Verbesina encelioides*	Asteraceae
BIR-BS	12	Birdsville	3/08/2007	−26.6707	139.0784	*Epaltes cunninghamii*	Asteraceae
SIM-BP1	19	Simpson	4/08/2007	−26.5743	137.2792	*Blennodia pterosperma*	Brassicaceae
SIM-GC	13	Simpson	4/08/2007	−26.5743	137.2792	*Goodenia cycloptera*	Goodeniaceae
SIM-SG1	13	Simpson	4/08/2007	−26.5742	137.2745	*Senecio gregorii*	Asteraceae
SIM-BP2	9	Simpson	5/08/2007	−26.3232	137.0422	*Blennodia pterosperma*	Brassicaceae
SIM-SG2	11	Simpson	5/08/2007	−26.3232	137.0422	*Senecio gregorii*	Asteraceae
SIM-CE	23	Simpson	6/08/2007	−25.9011	138.8192	*Crotalaria eremaea*	Fabaceae
SIM-CA	29	Simpson	7/08/2007	−25.9011	138.8192	*Cullen australasicum*	Fabaceae
EYR-CA	28	Eyre Creek	7/08/2007	−25.9002	138.8540	*Cullen australasicum*	Fabaceae
MIL-TS	29	Milparinka	10/08/2007	−29.5757	141.9153	*Trigonella suavissima*	Fabaceae
MIL-SG	26	Milparinka	10/08/2007	−29.5757	141.9153	*Swainsona galegifolia*	Fabaceae
MIL-SI	29	Milparinka	10/08/2007	−29.5757	141.9153	*Sisymbrium irio*	Brassicaceae
MIL-CP	17	Milparinka	10/08/2007	−29.5757	141.9153	*Cullen pallidum*	Fabaceae
TIL-EC	26	Tilpa	11/08/2007	−30.9365	144.4160	*Erodium cygnorum*	Geraniaceae
BOU-EC	31	Bourke	11/08/2007	−30.1743	145.8144	*Erodium cygnorum*	Geraniaceae
WAL-MP	26	Walget	11/03/2008	−30.0113	148.0635	*Malva parviflora*	Malvaceae
BRE-MP*	19	Brewarrina	11/03/2008	−29.9514	146.7633	*Malva parviflora*	Malvaceae
BRE-MS1*	25	Brewarrina	11/03/2008	−29.9621	146.8505	*Medicago sativa*	Fabaceae
BRE-EC*	26	Brewarrina	12/08/2007	−29.9520	146.7766	*Erodium cygnorum*	Geraniaceae
BRE-PC*	10	Brewarrina	12/08/2007	−29.9520	146.7766	*Phlegmatospermum cochlearinum*	Brassicaceae
BRE-MS2*	12	Brewarrina	12/08/2007	−29.9615	146.3504	*Medicago sativa*	Fabaceae
NAR-MP*	9	Narrabri	13/08/2007	−30.3256	149.7922	*Malva parviflora*	Malvaceae
NAR-GH*	24	Narrabri	20/01/2007	−30.2008	149.5724	*Gossypium hirsutum*	Malvaceae
NAR-MS*	23	Narrabri	20/01/2007	−30.2008	149.5724	*Medicago sativa*	Fabaceae

* indicate samples from agricultural sites.

### HWE, genetic diversity, genetic differentiation, and tests for recent bottlenecks

We estimated null allele frequency using the expectation maximization algorithm of Dempster et al. (1977) implemented in FreeNA (Chapuis and Estoup 2007) with 10,000 bootstrap resamplings. Deviations from Hardy–Weinberg Equilibrium (HWE) were calculated using the exact probability test (Guo and Thompson 1992) implemented in Genepop (Rousset 2008) and a sequential Bonferroni correction was applied per locus to account for multiple tests. Locus mirsat-3E showed deviations from HWE in many samples and was consequently shown to have relatively high frequencies of null alleles (Table 3) and was discarded. The total number of alleles per locus, average number of alleles per locus, and (Nei 1987) unbiased gene diversity (per locus and sample) were calculated using FSTAT (Goudet 2001). Expected (*H*e), observed (*H*o), and unbiased expected (*UH*e) heterozygosities were computed using Genalex (Peakall and Smouse 2006). Exact tests for linkage disequilibria were carried out in Genepop (Rousset 2008).

**Table 3:** Locus specific details for microsatellites used in this study; ∑*N*a, total number of alleles, 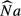, average number of alleles per population sampled, *H*o, observed, and He, expected heterozygosities, HWD (number of population samples deviating from Hardy–Weinberg Equilibrium), null allele frequencies, and locus specific global *F*_ST_ without and with the exclusion of null alleles

Locus	∑*N*a	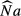	*H*o	*H*e	HWD	Null alleles	g*F*_ST_ Null	g*F*_ST_ No Null
mirsat-2F	11	4.88	0.52	0.56	2	0.051	0.17	0.17
mirsat-4B	10	3.61	0.31	0.46	2	0.080	0.27	0.25
mirsat-3E	16	5.97	0.25	0.56	13	0.199	0.21	0.18
mirsat-A1	9	2.52	0.19	0.26	5	0.074	0.19	0.21
mirsat-3H	21	5.48	0.41	0.42	1	0.048	0.09	0.08
mirsat-6B	6	1.76	0.03	0.09	0	0.067	0.03	0.09
mirsat-5C	20	7.94	0.82	0.77	0	0.034	0.04	0.04
mirsat-G8	13	4.67	0.30	0.54	6	0.174	0.03	0.03
mirsat-7G	15	5.91	0.69	0.68	2	0.038	0.06	0.06

The proportion of genetic variance that can be attributed to within-population comparisons and between-population comparisons was estimated using an analysis of molecular variance (AMOVA) in Genalex (Peakall and Smouse 2006). Unbiased pairwise and locus specific *F*_ST_'s (Weir 1996) were computed with and without the algorithm for the exclusion of null alleles (ENA) implemented in FreeNA (Chapuis and Estoup 2007). Pairwise exact tests of genotypic differentiation were computed using Genepop (Rousset 2008), as this estimator is more appropriate in situations where gene frequencies may deviate from HWE expectations, and a sequential Bonferroni adjustment was performed to account for multiple population comparisons.

To test for signatures of recent demographic bottlenecks in the microsatellite data, the Wilcoxon test for heterozygote excess (under the two-phase mutation model) and the allele frequency mode shift analysis were performed using the program BOTTLENECK (Piry et al. 1999) for all 32 populations, and a sequential Bonferroni adjustment applied.

### Spatiotemporal patterns of genetic differentiation and tests for migration

We tested for the presence of isolation by distance (IBD) to explore gene flow in relation to the temporal and geographic aspects of the sampling strategy. Initially, this analysis was restricted to the sampling period of July to August 2007 (when samples were widespread geographically and collected over a short period from both agricultural hosts and native inland hosts). Subsequently, all samples were analyzed to assess the temporal stability of the August 2007 pattern, these additional samples represented the same agricultural crops sampled in January 2007. The presence of an IBD effect was investigated by regressing ENA corrected genetic distance (*F*_ST_/(1−*F*_ST_)) against geographic distance (Rousset 1997). A Mantel test of matrix correspondence was used to test for significance using the Isolation By Distance Web Service (IBDWS) 3.15 (Jensen et al. 2005).

Patterns of genetic differentiation and admixture, which may be obscured by statistics that assume the correct a priori identification of populations, were clarified with the individual-based Bayesian clustering algorithm implemented in the program STRUCTURE (Pritchard et al. 2000). Low levels of null alleles are unlikely to affect the overall outcome of assignment testing such as the one implemented in the STRUCTURE algorithm (Carlsson 2008). The “admixture” model was used as the most appropriate for a species in which dispersal is likely. Initially, values of *K* from 1 to 14 were used, with a burn-in of 50,000 and a run length of 500,000, and each *K* value was replicated three times. These results were exported to the program STRUCTURE HARVESTER (Earl and VonHoldt 2011) and the most likely value of *K* for the data set was inferred using the Δ*K* method of Evanno et al. (2005). The data were then analyzed using this value for *K* with a burn-in of 100,000 and 1,000,000 subsequent iterations; this was replicated 10 times. The results were permuted with CLUMPP (Jakobsson and Rosenberg 2007) and the mean of the permuted results plotted using DISTRUCT (Rosenberg 2004).

Recent migration between arid inland sites and the eastern cropping regions was tested with the Bayesian assignment-based algorithm implemented in BAYESASS, which estimates rates of recent migration (within a few generations) from the proportion of individuals in each population sample that are assigned to other populations with high probability (Wilson and Rannala 2003). This algorithm does not assume that each designated population is in Hardy–Weinberg equilibrium, and produces reasonable estimates of actual migration in an experimental setting (Mardulyn et al. 2008). Furthermore, simulations indicate that the method provides fairly accurate estimates of *m* if genetic differentiation is not too low (*F*_ST_ ≥ 0.05) (Faubet et al. 2007), this is the case for all of the between-location comparisons used in our analyses. The results of the IBD test indicated that genetic differentiation is unstable across seasons, so we restricted our analysis of gene flow to estimators of recent migration rather than coalescent approaches to estimating long-term averages of migration such as MIGRATE (Beerli and Felsenstein 2001). Population samples were grouped by location and then split by the time of collection, because the results of the STRUCTURE analysis indicated significant temporal shifts in cluster assignment within sites. We ran the analyses for 3,000,000 iterations with a burn-in of 1,000,000 using the default delta value of 0.15 for allele frequency, migration rate, and inbreeding; the total log likelihood was plotted to assess convergence within runs. The computation was then performed 10 times with different starting seeds to assess convergence across runs by comparing the posterior probability densities of allele frequencies. The results of the 10 runs were converted to tabular format using a custom Perl script (available on request from the corresponding author) for comparison. The number of times each outcome was achieved over the 10 runs was recorded, and the mean migration rates were calculated for each of these outcomes. Migration rates with lower 95% confidence intervals below *m* = 0.02 were not considered significant and were also omitted. We used the lower 95% CI to assess the significance of migration rates because experimental tests in *Caenorhabditis remanei* indicate that actual migration rates tend to be lower than the inferred rates, but within the 95% CI (Mardulyn et al. 2008).

### Host plant-associated differentiation

Hierarchical AMOVA was performed across all 32 populations, with the higher order defined as host plant in Genalex (Peakall and Smouse 2006). The host plants in central Australia, however, are completely different species to those used by green mirids in eastern cropping regions. To test the hypothesis that differentiation might be associated with host plant usage, we tested for genetic differentiation across host plants with respect to two inland localities within which multiple host plants had been sampled (namely Simpson Desert and Milparinka). The STRUCTURE algorithm was run using the admixture model with a burn-in of 100,000 and 500,000 subsequent iterations, with *K* = 2 “population” clusters. The most likely value of *K* was determined by the delta *K* method (Evanno et al. 2005) implemented in STRUCTURE HARVESTER (Earl and VonHoldt 2011).

## Results

### Mitochondrial

Genetic diversity was low for the mitochondrial COI sequences, with one haplotype dominating each of the three temporal samples. The 2006/2007 samples (*n* = 146) had a nucleotide diversity (π) of 0.00055, and a haplotype diversity of 0.278. Tajima's D was −2.26 (*P* < 0.01) indicating an excess of low frequency polymorphisms likely due to population expansion. This pattern was similar in 1983 (*n* = 16), where nucleotide diversity was 0.00021, haplotype diversity 0.125, and Tajima's D −1.16, but not significant (*P* > 0.10), and in 1993 (*n* = 25), π = 0.00026, haplotype diversity 0.153, and Tajima's D −0.69, also not significant (*P* > 0.10).

**Figure 1:**
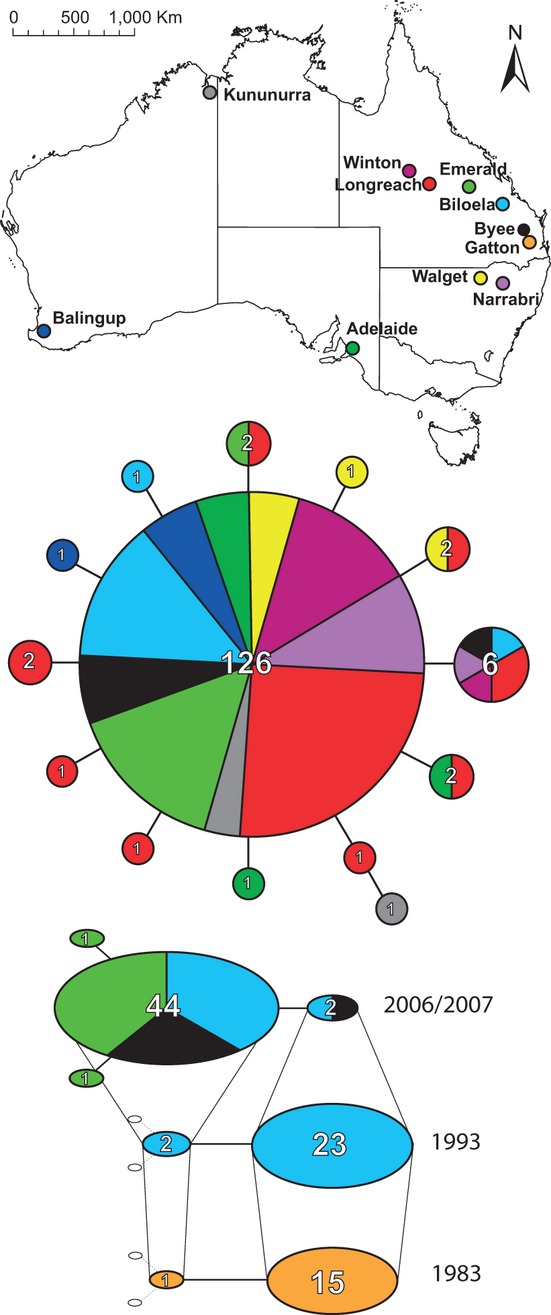
Map (top) showing sampling locations for the COI sequences obtained for this study (location colors maintained throughout the figure). Haplotype network (middle) showing all sequences from 2006/2007, and temporal haplotype network (bottom) showing the change in haplotype frequencies in Eastern Queensland between 1983 and 2006/2007. The area of each haplotype (circles and ellipses) represents the number of individuals having that haplotype (numbers inside haplotypes), empty ellipses (bottom figure) show haplotypes present in the 2006/2007 samples, but not in 1993 or 1983.

In the eastern cropping regions of Queensland, where a comparison of haplotype frequencies could be made across three temporal samples spanning 24 years, there has been a shift in the dominant haplotype between 1993 and the more recent samples (Fig. 1, bottom). The haplotype that was prevalent across the whole of Australia in 2006/2007 (Fig. 1 middle) was present at much lower frequency in the earlier samples (Gatton, 1983 and Biloela 1993) where the same haplotype was predominant despite a distance of 400 km between these two sites. The Biloela samples (1993 and 2006/2007) were all collected from the Queensland Government Biloela Research station (Table 1).

### HWE, genetic diversity, genetic differentiation, and tests for recent bottlenecks

**Figure 2:**
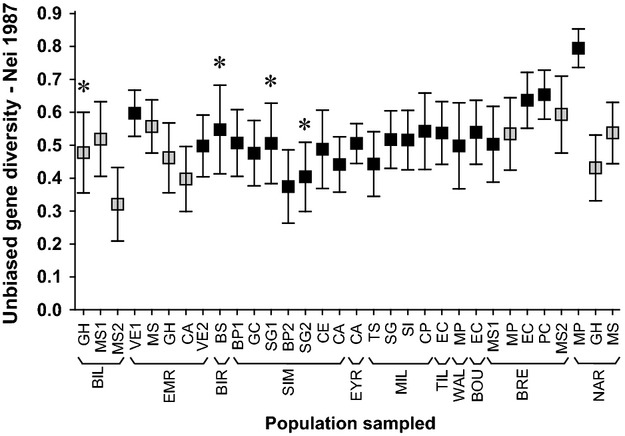
Nei's unbiased gene diversity averaged across loci for all populations. Gray boxes represent samples collected from agricultural crops; black boxes represent samples collected from non-crop hosts. Asterisks indicate population samples for which there is some evidence of a recent bottleneck (see Results for details).

A total of 105 alleles weresscored across all loci and all populations, once the null allele prone locus mirsat-3E had been removed (Table 3). Unbiased gene diversity for each population (Nei 1987), when averaged across loci, ranged between 0.32 and 0.79 (mean 0.51) and was not significantly different between samples from agriculture and those taken inland (Fig. 2, two-tailed permutation test, *P* = 0.275). Four of 32 population samples, three from inland and one from agriculture, showed genetic signatures indicating a recent bottleneck in the allele mode shift analysis (BIR-BS, SIM-SG1, SIM-SG2, BIL-GH, Fig. 2), although only two of these showed a significant heterozygote excess in the Wilcoxon test (BIR-BS, *P* = 0.0117 and BIL-GH, *P* = 0.0078). In addition, three of the four populations showed indications of admixture, and neither of the Wilcoxon tests was significant (with an alpha probability of *P* > 0.05 after Bonferroni correction).

Deviations from HWE were inferred in all loci for some populations, and the presence of null alleles was also inferred (Table 3). HWE deviations might, however, be expected in recently admixed populations due to the Wahlund effect. We took three approaches to assess and minimize the effects of null alleles: 1) estimation of *F*_ST_ values using null allele corrected and non-corrected data; 2) removal of the two loci that had the greatest effect on HWE (mirsat1A1 and mirsat2G8), and then comparing results across the 6 locus and 8 locus data sets; and 3) selection of analyses that are more robust to low frequencies of null alleles and small deviations from HWE (see methods for details). Evaluating the effects of null allele/HWE deviations using these three methods revealed that the low frequencies of null alleles inferred in some population samples for some loci did not dramatically affect the overall signal in the data, and all results shown are for the 8-locus data set. Tests for genotypic linkage disequilibria returned no significant associations between pairs of loci for any of the 32 population samples after sequential Bonferroni correction for multiple comparisons.

The AMOVA apportioned 19% of all molecular variance to among-population comparisons (*Φ*_PT_ = 0.188, *P* = 0.001). The global *F*_ST_ estimates were similar with or without the elimination of null alleles, with the uncorrected data returning only a slightly higher estimate (*F*_ST_ = 0.122 using the ENA algorithm and 0.128 without ENA correction). Pairwise *F*_ST_'s ranged from 0.0019 to 0.329 (mean = 0.112), with 374 of 528 comparisons of genotypic differentiation being significant after sequential Bonferroni correction (Table S1).

### Spatiotemporal patterns of genetic differentiation and tests for migration

**Figure 3:**
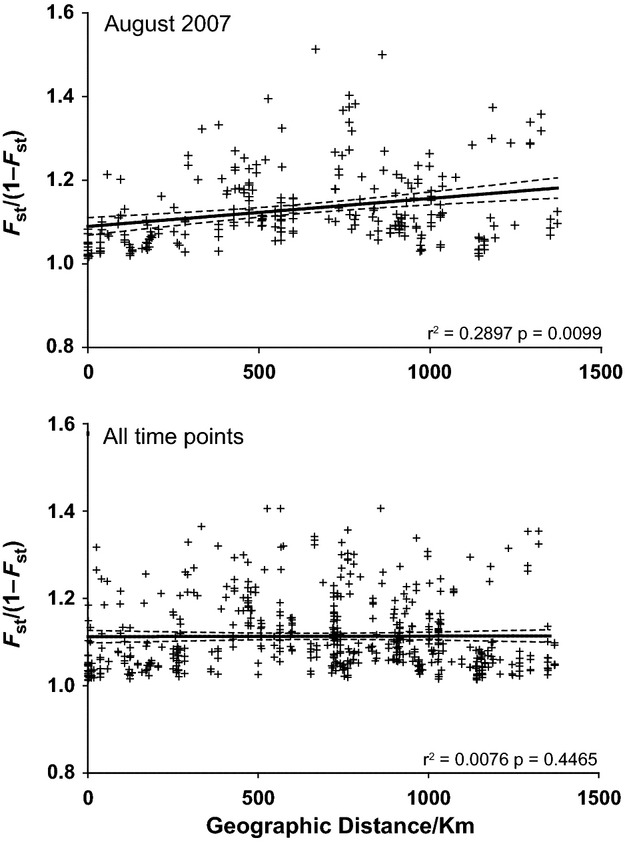
Results of Mantel test for isolation by distance. Above: Samples collected during August 2007 (*r* = 0.2897, *P* = 0.0099). Below: All samples collected (January 2007–March 2008) (*r* = 0.0076, *P* = 0.4465). Solid line shows the matrix correlations, and dashed lines show 95% confidence intervals.

The Mantel test of correspondence between geographic distance and genetic differentiation revealed a significant isolation by distance pattern when the analysis was restricted to the broad-scale geographic sampling of July to August 2007 (Fig. 3, *r* = 0.2897, *P* = 0.0099). In contrast, when all sampling events were included in the analysis (January 2007–March 2008), no isolation by distance effect was evident (*r* = 0.0076, *P* = 0.4465). The inclusion of these additional samples represented the same host plants that were sampled in agricultural regions during the July–August 2007 collections.

**Figure 4:**
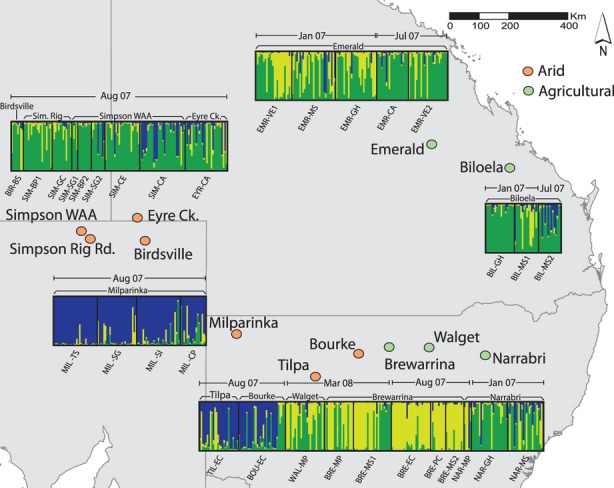
Results of STRUCTURE clustering analysis, separated into blocks showing the geographic origin and date of sampling. Each bar represents one individual; the proportion of each color represents the posterior probability of assignment to one of three clusters.

The Δ*K* method (Evanno et al. 2005) indicated that *K* = 3 was the most likely number of genetic clusters for this data set. The combined and permuted results of the subsequent 10 runs of the STRUCTURE algorithm with *K* set at 3 are shown in relation to the geographic origin of the population samples (Fig. 4). Individuals from Milparinka, Tilpa, and Bourke are mostly assigned to one cluster with high posterior probabilities, and these populations yielded the highest pair-wise *F*_ST_ values when compared with the other sites (*F*_ST_ = 0.047–0.307; mean = 0.132; 148 of 156 tests of genotypic differentiation significant). Admixture was evident in several populations, as several individuals had mixed posterior probabilities of assignment to each of the other two clusters. The proportion of admixed individuals and their cluster assignment shifted between January 2007 and July 2007 in both Biloela and Emerald, and between January 2007 and August 2007 in Narrabri (Fig. 4.)

**Figure 5:**
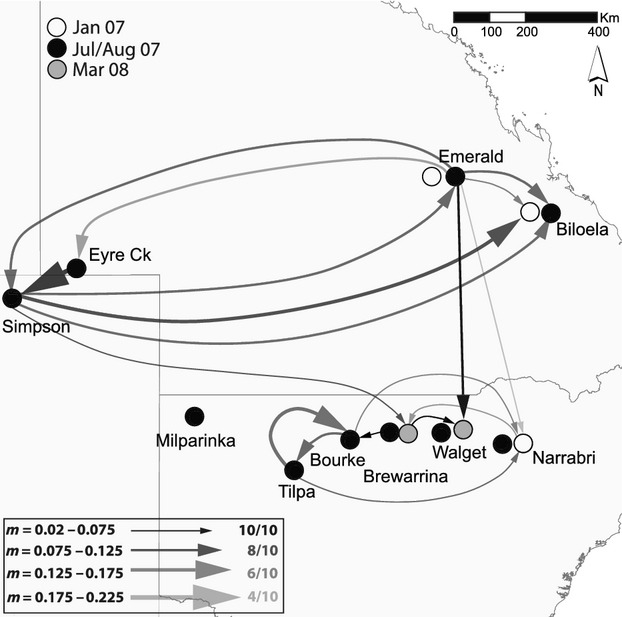
Graphical representation of migration rates inferred using the Bayesian assignment algorithm in BayesAss. The size of the arrows indicates the migration rate (*m*), whereas the shade of the arrows indicates the number of times this outcome was reached over 10 runs with varying starting seeds. Outcomes that were reached less than four times, and migration rates lower than 0.02 are not shown. (See text and Table S2, supporting information, for 95% CI's).

We detected significant levels of recent migration, at the full geographic extent of sampling, using the BAYESASS algorithm. Variability was detected across runs of the algorithm, but this was characterized as a reversal of the inferred direction of migration between sites rather than changes in the sites between which dispersal was inferred. Figure 5 is a graphical representation of the migration rates and the frequency that each migration outcome was reached over 10 runs of the algorithm using different starting seed (See Table S2 for the full results, including 95% CI's). Significant migration was inferred between the Simpson Desert sites, in the arid interior, and the sub-coastal agricultural areas in Queensland (Biloela and Emerald), for example, Simpson (August 2007) to Biloela (January 2007), *m* = 0.14, lower 95% CI = 0.08, upper 95% CI = 0.21, 6/10 runs. Although the direction of migration was most often toward agricultural regions, the direction was not always consistent across runs and strong inference cannot be made as to the direction of dispersal from this result. Migration between Milparinka and other sites was not inferred from the genetic data, which is consistent with the outcomes of the STRUCTURE analysis and pairwise *F*_ST_'s.

### Host plant-associated differentiation

**Figure 6:**
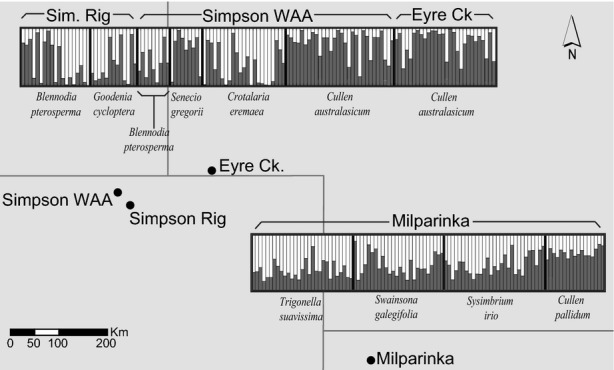
STRUCTURE analysis restricted to sites where multiple host plants were present and sampled. Each bar represents the posterior probability that the individual belongs to one of two clusters. The host plant species from which the particular samples were collected is listed below each population.

The Hierarchical AMOVA indicated an effect of host plant on molecular variance (*Φ*_RT_ = 0.078, *P* = 0.001); however, host plant species were not sampled consistently across the whole of the sampling area (because each has a restricted distribution relative to the scale of the study). Genetic differentiation in relation to host plant species was therefore evaluated across two sites where several species could be sampled at each. At both of these, some degree of genetic differentiation was associated with plants in the genus *Cullen* relative to all other host plants sampled, although this was more pronounced at the Simpson Desert sites than at Milparinka in western New South Wales (Fig. 6) (but note that at Milparinka, *C. dilutus* was sampled from *Cu. pallidum* and at the Simpson Desert/Eyre creek sites from *Cu. australasicum*). The Evanno et al. (2005) delta *K* method indicated that *K* = 2 was the most likely number of clusters for the Simpson site, but there was no peak in delta *K* for the Milparinka site. Further examination of the genotyping data at these two sites revealed that the genetic differentiation indicated by the STRUCTURE analysis appears to stem from the higher occurrence of rare alleles on *Cullen* host plants than on alternative hosts.

## Discussion

Our combined analyses of genetic patterns in green mirid populations indicate that they are likely shaped by changes in population size (Fig. 1), admixture (Fig. 4), and recent migration (Fig. 3, Fig. 6). Our results are consistent with the expectations of complex spatiotemporal dynamics (Whitlock 1992; Wegmann et al. 2006). These dynamics are likely to arise from the extreme spatial and temporal environmental heterogeneity that typify dry years in this bug's native range in arid Australia. Heterozygote excesses and allele frequency mode shifts indicate that several populations may have passed through recent localized bottlenecks, although alternate processes can cause heterozygote excess. However, the prevalence of a single COI haplotype across the whole of the continent (from 2006/2007 samples), negative Tajima D values, low haplotype diversity in the older samples, and the shift in the most prevalent haplotype between 1993 and 2006/2007 (Fig. 1) are consistent with recurrent reductions in population size over a longer period. These past reductions in population size may reflect alternate periods of drought (when resources are ephemeral and spatially patchy) and floods, which cause widespread environmental homogeneity in terms of host plant availability. The present broad geographic prevalence of the one COI haplotype, inference of recent migration from the microsatellite data (Fig. 5), and admixture in agricultural populations (Fig. 4) demonstrate that populations in arid and agricultural regions are connected by gene flow. Although weak genetic differentiation was detected locally (within arid regions) across their primary host plants (in the genus *Cullen*) and alternative plant species, it was mostly the presence of rare alleles that was responsible for this pattern. We suggest that rare alleles on the primary hosts (*Cu. cinereum* and *Cu. australasicum*) could be a consequence of higher abundance of *C. dilutus* relative to their abundance on alternative host species. These points are expanded and justified below.

### Genetic diversity and founder effects

We assessed genetic diversity and possible founder effects across inland and agricultural sites to determine whether ephemeral host availability (inland) or pesticide use (in agriculture) affected the temporal stability of patterns of genetic differentiation between mirid populations. We found no significant difference in microsatellite diversity between *C. dilutus* collected from crop hosts and those collected from non-crop hosts (Fig. 2). Genetic signals of recent bottleneck events (heterozygote excess) were present in the microsatellite data in three inland and one agricultural population of *C. dilutus* (Fig. 2). None of the tests, however, were significant after Bonferroni correction, so we can only tentatively infer localized contractions. Lucerne, the primary crop host of *C. dilutus*, is often grown without pesticides in Australia, and insecticide-induced bottlenecks are less likely to be driving patterns of genetic differentiation than the arid dynamics of this mirid species.

Genetic differentiation among *C. dilutus* populations was higher than generally reported for pest insect species surveyed within an agricultural context (Endersby et al. 2006, 2007; Kim et al. 2009; Torres and Azeredo-Espin 2009). The highest *F*_ST_ values were attributed to pair-wise comparisons among three arid inland sites in New South Wales (Milparinka, Tilpa, and Bourke), and other populations. The Structure analysis also clearly differentiated populations at these three sites from others. Although no heterozygote excess was detected at these sites (indicative of a recent bottleneck), we suspect that a combination of spatial heterogeneity and founder effects could contribute to strong genetic drift (and therefore high genetic differentiation) given that our study was conducted during a dry period when patches of host plants were separated by large areas of barren land. Elevated *F*_ST_'s are predicted (even when migration rates are high) under spatiotemporally dynamic population models if environmental heterogeneity contributes to a large variance in local population size (Wegmann et al. 2006), a scenario consistent with the ecology of *C. dilutus*.

The low nucleotide diversity in both the older and more recent samples (2006/2007 π = 0.00055, 1983 = 0.00021, 1993 = 0.00026) and change in predominant mitochondrial haplotype over the last 13 years was striking. Mitochondrial DNA is expected to suffer a more extreme loss of alleles than nuclear markers during demographic bottlenecks due to the uniparental inheritance of the plastid and the reduced effective population size of its genome (Wilson et al. 1985; Simon et al. 1994). For example, a local population founded by a single gravid female would have one mitochondrial haplotype, but potentially four microsatellite alleles. Similar shallow “star shaped” genealogies and negative values of Tajima's D have been reported in agriculturally damaging insects with documented dispersal capacity (Albernaz et al. 2012), and in the case of the widespread noctuid pest *Helicoverpa armigera,* this pattern even spans continents (Behere et al. 2007). Human assisted range expansion of pest insects through the provision of agricultural resources is the scenario that typically explains widespread haplotypes (Grapputo et al. 2005). In green mirids, however, the dominant haplotype not only occurs across both agricultural and native arid regions but also has changed within the last 24 years, indicating that the alternation between dry and wet years in arid regions could be responsible, rather than the introduction of agricultural resources over the last 200 years. The low haplotype diversity in the older samples indicates that at least two such population contractions are likely to have occurred.

### Long-distance dispersal between arid and agricultural populations

That one haplotype is now dominant across the 5000-km width of Australia indicates that dispersal in *C. dilutus* has been widespread. Geographic differentiation was higher in the microsatellite data set, but the geographic distribution was not stable over time. This is evident from the temporary nature of the isolation by distance effect (Fig. 3), the temporal shifts in cluster assignment in the STRUCTURE analysis at Biloela, Emerald, and Narrabri (Fig. 4), and by the change in most prevalent COI haplotype between 1993 and 2006. Admixture across large geographic distances most likely results from dispersal, and this is evidenced by the inference (BAYESASS; Fig. 5) of significant migration rates across distances over 1500 km. The direction of inferred migration was not consistent across multiple runs of the algorithm, so conclusions regarding the directionality of dispersal remain tentative. Return migration from agricultural regions back to Central Australia by pest populations that derived originally from central desert areas (as postulated for *Helicoverpa punctigera*, which is also an Australian arid adapted species) is thought to be unlikely based on prevailing wind directions and because positive evidence of its existence has never been found (Downes et al. 2010).

The sites between which migration was inferred were consistent across runs of the BayesAss algorithm, and are thus likely to represent sites between which recent migration has occurred. *Creontiades dilutus* populations can expand rapidly, and abundance is seasonally inverse between inland and eastern regions. A migration event in late spring/early summer, when numbers are high in inland areas and low in cropping regions, might therefore result in a much higher inferred migration rate than the actual number of individuals migrating and establishing successfully. The regular seasonal influx of *C. dilutus* to cotton crops, which does not appear to be derived from local lucerne populations (Miles 1995), indicates that dispersal from inland populations may be a regular occurrence, the microsatellite data support this hypothesis, but it does require further direct testing.

### Host plant-associated genetic differentiation in arid regions

We found weak genetic differentiation between *C. dilutus* from *Cu. australasicum* and alternative hosts in the same geographic area (Simpson) in the structure analysis (Fig. 6), which may partly account for the significant role of host plants implicated by the hierarchical AMOVA. Plants in the genus *Cullen* maintain a significantly higher density of *C. dilutus* than other available hosts, indicating that plants in this genus are primary hosts for green mirids (Hereward and Walter 2012). However, analyses of gut contents using chloroplast intron markers revealed that a substantial proportion of *C. dilutus* individuals collected from the *Cullen* primary host plants had recently fed on other host plants (Hereward and Walter 2012). The use of multiple plant species by *C. dilutus* is perhaps best understood as a behavioral adaptation to survive in an arid environment where host plants are ephemeral and the primary host species not always available (Velasco and Walter 1993). The physiological and behavioral processes that underpin their multiple host use warrant investigation in association with the movement of individuals.

Previous quantified sampling showed that *C. dilutus* abundance was significantly higher on the *Cullen* hosts, *Cu. cinereum* and *Cu. australasicum*, than alternative hosts locally, but not *Cu. pallidum* (Hereward and Walter 2012). We detect genetic differentiation in green mirids between *Cu. australasicum* and other hosts locally, but not for *Cu. pallidum* (Fig. 6), although this pattern requires further replication to ensure that it is not the result of fine scale spatial differentiation. More rare microsatellite alleles were present in green mirid populations from *Cu. australasicum* than from alternative hosts. This may be a consequence of a much greater proportion of green mirids being attracted to these plants (perhaps from refuges provided by alternate hosts) and surviving. Further temporal samples at these inland sites would allow temporal estimates of effective population size across primary and secondary host plants, and the relative absence (and perhaps even loss) of rare alleles on alternative hosts needs to be investigated directly if these patterns are to be understood mechanistically.

### Conclusions and implications

*Creontiades dilutus* shows evidence of widespread dispersal in both the mitochondrial and microsatellite data sets examined here, despite this species using different plant resources (both locally and regionally), having seasonally inverse abundance between inland and agricultural regions, and presumably experiencing different selective pressures in these regions of Australia. The change in the most prevalent mitochondrial haplotype over 24 years is consistent with successive population contractions and expansions, likely in relation to fluctuations between dry periods and wet periods in the arid regions of Australia. Dispersal appears to be the major mechanism by which *C. dilutus* is able to survive on the ephemeral resources in this region, and the data provide no indication that large numbers of these bugs persist through dry periods by diapause. The spatiotemporal dynamics and changing gene frequencies outlined above contrast with the lack of genetic differentiation found in the same agricultural regions over several years for the highly dispersing *H. armigera* (Endersby et al. 2007) and the temporal stability of allele frequencies recoded for Queensland fruit fly (Yu et al. 2001). These dynamics also differ from the stepwise founder effects associated with insects that colonize new temporally stable habitat “islands” through human movement (Stone and Sunnucks 1993). Patterns of genetic differentiation and gene flow in green mirids seem to be driven instead by the spatial and temporal heterogeneity of their native hosts, but these same effects have spread to agricultural regions. This fits with Oliver (Oliver 2006)'s hypothesis that the expansion of host resources is likely to increase gene flow in native insects.

With such spatiotemporal dynamics, adaptation to novel host plants is unlikely. We nevertheless found weak host-associated differentiation between green mirids on their primary host plants and those on alternative hosts growing locally, despite establishing previously that these individuals will feed on alternative host species even when in the nearby vicinity of the primary host (Hereward and Walter 2012). Many herbivorous insects that use multiple hosts have been shown, by thorough quantitative sampling, to have a similar closer affinity to one host species than others that it may use (Milne and Walter 2000; Rajapakse et al. 2006; Manners and Walter 2009). Assessing gene flow and genetic diversity in many of these instances might further our understanding of multiple host use by herbivorous insects.

Our results highlight the importance of assessing evolutionary and ecological processes across the distribution of an organism that uses both native and human-altered habitats simultaneously. If our analyses had been restricted to either agricultural areas or localized parts of the arid range of this species, our interpretations might be quite different. For example, broader geographic analyses of *Rhagoletis pomonella*, perhaps the most famous example of host-associated differentiation following the human introduction of novel hosts (cultivated apple) (Bush 1993), to include native hosts in Mexico, indicates that the differences in host plant use had an allopatric rather than sympatric origin (Feder et al. 2003; Michel et al. 2007). In *C. dilutus,* we find that the spatiotemporal dynamics in its arid native range continue to drive genetic patterns across both arid and agricultural environments. The adaptations that allow it to persist despite the spatio-temporal heterogeneity of host resources in arid regions (migration and the use of alternative hosts) appear to have not only facilitated the colonization of new agricultural habitats but also maintain gene flow across large distances.

## Supporting Information

**Table S1.** Pairwise *F*_ST_'s for all sites based on the microsatellite data.

**Table S2.** Results of 10 runs of the BayesAss Algorithm, with averages and total number of times the result was achieved.

## References

[b1] Albernaz K, Silva-Brandão K, Fresia P, Cônsoli F, Omoto C (2012). Genetic variability and demographic history of Heliothis virescens (Lepidoptera: Noctuidae) populations from Brazil inferred by mtDNA sequences. Bull. Entomol. Res.

[b2] Andris M, Aradottir GI, Arnau G, Audzijonyte A, Bess EC, Bonadonna F (2010). Permanent Genetic Resources added to Molecular Ecology Resources Database 1 June 2010–31 July 2010. Mol. Ecol. Resour.

[b4] Beerli P, Felsenstein J (2001). Maximum likelihood estimation of a migration matrix and effective population sizes in n subpopulations by using a coalescent approach. Proc. Natl Acad. Sci.

[b5] Behere G, Tay W, Russell D, Heckel D, Appleton B, Keshav K (2007). Mitochondrial DNA analysis of field populations of Helicoverpa armigera (Lepidoptera: Noctuidae) and of its relationship to H. zea. BMC Evol. Biol.

[b6] Bohonak AJ (1999). Dispersal, gene flow, and population structure. Q. Rev. Biol.

[b7] Bush G (1993). Host race formation and speciation in Rhagoletis fruit flies (Diptera: Tephritidae). Psyche.

[b8] Carlsson J (2008). Effects of microsatellite null alleles on assignment testing. J. Hered.

[b9] Chapuis MP, Estoup A (2007). Microsatellite null alleles and estimation of population differentiation. Mol. Biol. Evol.

[b10] Chapuis MP, Lecoq M, Michalakis Y, Loiseau A, Sword GA, Piry S (2008). Do outbreaks affect genetic population structure? A worldwide survey in Locusta migratoria, a pest plagued by microsatellite null alleles. Mol. Ecol.

[b11] Chapuis M-P, Loiseau A, Michalakis Y, Lecoq M, Franc A, Estoup A (2009). Outbreaks, gene flow and effective population size in the migratory locust, Locusta migratoria: a regional-scale comparative survey. Mol. Ecol.

[b12] Dempster AP, Laird NM, Rubin DB (1977). Maximum likelihood from incomplete data via EM algorithm. J. Roy. Statist. Soc.Ser. B Stat. Methodol.

[b13] Downes S, Parker T, Mahon R (2010). Incipient Resistance of *Helicoverpa punctigera* to the Cry2Ab Bt Toxin in Bollgard II® Cotton. PLoS ONE.

[b14] Drury DW, Siniard AL, Wade MJ (2009). Genetic differentiation among wild populations of tribolium castaneum estimated using microsatellite markers. J. Hered.

[b15] Earl DA, VonHoldt BM (2011). STRUCTURE HARVESTER: a website and program for visualizing STRUCTURE output and implementing the Evanno method. Conserv. Genet. Resour.

[b16] Endersby NM, McKechnie SW, Ridland PM, Weeks AR (2006). Microsatellites reveal a lack of structure in Australian populations of the diamondback moth, *Plutella xylostella* (L.). Mol. Ecol.

[b17] Endersby NM, Hoffmann AA, McKechnie SW, Weeks AR (2007). Is there genetic structure in populations of *Helicoverpa armigera* from Australia?. Entomol. Exp. Appl.

[b18] Evanno G, Regnaut S, Goudet J (2005). Detecting the number of clusters of individuals using the software STRUCTURE: a simulation study. Mol. Ecol.

[b19] Faubet P, Waples RS, Gaggiotti OE (2007). Evaluating the performance of a multilocus bayesian method for the estimation of migration rates. Mol. Ecol.

[b20] Feder JL, Berlocher SH, Roethele JB, Dambroski H, Smith JJ, Perry WL (2003). Allopatric genetic origins for sympatric host-plant shifts and race formation in Rhagoletis. Proc. Natl Acad. Sci.

[b21] Folmer O, Black M, Hoeh W, Lutz R, Vrijenhoek R (1994). DNA primers for amplification of mitochondrial cytochrome c oxidase subunit I from diverse metazoan invertebrates. Mol. mar. biol.

[b22] Franklin MT, Ritland CE, Myers JH (2010). Spatial and temporal changes in genetic structure of greenhouse and field populations of cabbage looper, *Trichoplusia ni*. Mol. Ecol.

[b23] Funk DJ, Filchak KE, Feder JL (2002). Herbivorous insects: model systems for the comparative study of speciation ecology. Genetica.

[b24] Gaete-Eastman C, Figueroa CC, Olivares-Donoso R, Niemeyer HM, Ramírez CC (2007). Diet breadth and its relationship with genetic diversity and differentiation: the case of southern beech aphids (Hemiptera: Aphididae). Bull. Entomol. Res.

[b25] Goudet J (2001). http://www2.unil.ch/popgen/softwares/fstat.htm.

[b26] Grapputo A, Boman S, Lindstrom L, Lyytinen A, Mappes J (2005). The voyage of an invasive species across continents: genetic diversity of North American and European Colorado potato beetle populations. Mol. Ecol.

[b27] Groot A, Classen A, Inglis O, Blanco C, Lopez J, Teran Vargas A (2011). Genetic differentiation across North America in the generalist moth Heliothis virescens and the specialist H. subflexa. Mol. Ecol.

[b28] Guo SW, Thompson EA (1992). Performing the exact test of Hardy-Weinberg proportion for multiple alleles. Biometrics.

[b29] Habel J, Meyer M, Schmitt T (2009). The genetic consequence of differing ecological demands of a generalist and a specialist butterfly species. Biodiversity Conserv.

[b30] Hereward JP, Walter GH (2012). Molecular interrogation of the feeding behaviour of field captured individual insects for interpretation of multiple host plant use. PLoS ONE.

[b31] Jakobsson M, Rosenberg NA (2007). CLUMPP: a cluster matching and permutation program for dealing with label switching and multimodality in analysis of population structure. Bioinformatics.

[b32] Jensen JL, Bohonak AJ, Kelley ST (2005). Isolation by distance, web service. BMC Genet.

[b33] Kelley S, Farrell B, Mitton J (2000). Effects of specialization on genetic differentiation in sister species of bark beetles. Heredity.

[b34] Khan M, Gregg P, Mensah R (2009). Effect of temperature on the biology of *Creontiades dilutus* (Stal) (Heteroptera: Miridae). Aust. J. Entomol.

[b35] Kim KS, Bagley MJ, Coates BS, Hellmich RL, Sappington TW (2009). Spatial and Temporal Genetic Analyses Show High Gene Flow Among European Corn Borer (Lepidoptera: Crambidae) Populations Across the Central US Corn Belt. Environ. Entomol.

[b36] Kobayashi T, Sakurai T, Sakakibara M, Watanabe T (2011). Multiple origins of outbreak populations of a native insect pest in an agro-ecosystem. Bull. Entomol. Res.

[b37] Letnic M, Dickman C (2006). Boom means bust: interactions between the El Niño/Southern Oscillation (ENSO), rainfall and the processes threatening mammal species in arid Australia. Biodiversity Conserv.

[b38] Li J, Li H, Jakobsson M, Li SEN, Sjodin PER, Lascoux M (2012). Joint analysis of demography and selection in population genetics: where do we stand and where could we go?. Mol. Ecol.

[b39] Librado P, Rozas J (2009). DnaSP v5: a software for comprehensive analysis of DNA polymorphism data. Bioinformatics.

[b40] Malipatil MB, Cassis G (1997). Taxonomic review of Creontiades distant in Australia (Hemiptera: Miridae: Mirinae). Aust. J. Entomol.

[b41] Manners AG, Walter GH (2009). Multiple host use by a sap-sucking membracid: population consequences of nymphal development on primary and secondary host plant species. Arthropod Plant Interact.

[b42] Mardulyn P, Vaesen MA, Milinkovitch MC (2008). Controlling population evolution in the laboratory to evaluate methods of historical inference. PLoS ONE.

[b43] Margaritopoulos J, Kasprowicz L, Malloch G, Fenton B (2009). Tracking the global dispersal of a cosmopolitan insect pest, the peach potato aphid. BMC Ecol.

[b44] McColl SA, Khan M, Umina PA (2011). Review of the biology and control of Creontiades dilutus (Stal) (Hemiptera: Miridae). Aust. J. Entomol.

[b45] Medina RF, Reyna SM, Bernal JS (2012). Population genetic structure of a specialist leafhopper on Zea: likely anthropogenic and ecological determinants of gene flow. Entomol. Exp. Appl.

[b46] Michel AP, Rull J, Aluja M, Feder JL (2007). The genetic structure of hawthorn-infesting Rhagoletis pomonella populations in Mexico: implications for sympatric host race formation. Mol. Ecol.

[b47] Miles MM (1995). Identification, Pest Status, Ecology and Management of the Green Mirid, Creontiades dilutus (Stal) (Hemiptera: Miridae), a Pest of Cotton in Australia.

[b48] Miller SA, Dykes DD, Polesky HF (1988). A simple salting out procedure for extracting DNA from human nucleated cells. Nucleic Acids Res.

[b49] Milne M, Walter GH (2000). Feeding and breeding across host plants within a locality by the widespread thrips Frankliniella schultzei, and the invasive potential of polyphagous herbivores. Divers. Distrib.

[b50] Morton SR, Smith DMS, Dickman CR, Dunkerly DL, Friedel MH, McAllister RRJ (2011). A fresh framework for the ecology of arid Australia. J. Arid Environ.

[b51] Nei M (1987). Molecular evolutionary genetics.

[b52] Nicholls N (1991). The El Niño/Southern oscillation and Australian vegetation. Plant Ecol.

[b53] Oliver JC (2006). Population genetic effects of human-mediated plant range expansions on native phytophagous insects. Oikos.

[b54] Pavlidis P, Hutter S, Stephan W (2008). A population genomic approach to map recent positive selection in model species. Mol. Ecol.

[b55] Peakall R, Smouse PE (2006). GENALEX 6: genetic analysis in Excel. Population genetic software for teaching and research. Mol. Ecol. Notes.

[b56] Piry S, Luikart G, Cornuet JM (1999). BOTTLENECK: A computer program for detecting recent reductions in the effective population size using allele frequency data. J. Hered.

[b57] Pritchard JK, Stephens M, Donnelly P (2000). Inference of population structure using multilocus genotype data. Genetics.

[b58] Prost S, Anderson C (2012). http://www.stanford.edu/group/hadlylab/tempnet/.

[b59] Rajapakse CNK, Walter GH, Moore CJ, Hull CD, Cribb BW (2006). Host recognition by a polyphagous lepidopteran (Helicoverpa armigera): primary host plants, host produced volatiles and neurosensory stimulation. Physiol. Entomol.

[b60] Ridley A, Hereward J, Daglish G, Raghu S, Collins PJ, Walter GH (2011). The spatiotemporal dynamics of Tribolium castaneum (Herbst): adult flight and gene flow. Mol. Ecol.

[b61] Rosenberg NA (2004). DISTRUCT: a program for the graphical display of population structure. Mol. Ecol. Notes.

[b62] Rousset F (1997). Genetic differentiation and estimation of gene flow from F-statistics under isolation by distance. Genetics.

[b63] Rousset F (2008). GENEPOP ‘007: a complete re-implementation of the GENEPOP software for Windows and Linux. Mol. Ecol. Resour.

[b64] Semeao AA, Campbell JF, Beeman RW, Lorenzen MD, Whitworth RJ, Solderbeck PE (2012). Genetic Structure of Tribolium castaneum (Coleoptera: Tenebrionidae) Populations in Mills. Environ. Entomol.

[b65] Simon C, Frati F, Beckenbach A, Crespi B, Liu H, Flook P (1994). Evolution, weighting, and phylogenetic utility of mitochondrial gene sequences and a compilation of conserved polymerase chain reaction primers. Ann. Entomol. Soc. Am.

[b66] Stone G, Sunnucks P (1993). Genetic consequences of an invasion through a patchy environment the cynipid gallwasp Andricus quercuscalicis (Hymenoptera: Cynipidae). Mol. Ecol.

[b67] Stone G, Challis R, Atkinson R, Csoka G, Hayward A, Melika G (2007). The phylogeographical clade trade: tracing the impact of human-mediated dispersal on the colonization of northern Europe by the oak gallwasp Andricus kollari. Mol. Ecol.

[b68] Sword GA, Joern A, Senior LB (2005). Host plant-associated genetic differentiation in the snakeweed grasshopper, Hesperotettix viridis (Orthoptera: Acrididae). Mol. Ecol.

[b69] Torres TT, Azeredo-Espin AML (2009). Population genetics of New World screwworm from the Caribbean: insights from microsatellite data. Med. Vet. Entomol.

[b70] Velasco LRI, Walter GH (1993). Potential of host-switching in *Nezara viridula* (Hemiptera, Pentatomidae) to enhance survival and reproduction. Environ. Entomol.

[b71] Via S (1990). Ecological genetics and host adaptation in herbivorous insects - the experimental study of evolution in natural and agricultural systems. Annu. Rev. Entomol.

[b72] Voudouris C, Franck P, Olivares J, Sauphanor B, Mamuris Z, Tsitsipis J (2012). Comparing the genetic structure of codling moth Cydia pomonella (L.) from Greece and France: long distance gene-flow in a sedentary pest species. Bull. Entomol. Res.

[b73] Waples RS, Teel DJ (1990). Conservation genetics of pacific salmon I. temporal changes in allele frequency. Conserv. Biol.

[b74] Wegmann D, Currat M, Excoffier L (2006). Molecular diversity after a range expansion in heterogeneous environments. Genetics.

[b75] Weir BS (1996). Genetic Data Analysis II.

[b76] Whitlock MC (1992). Temporal fluctuations in demographic parameters and the genetic variance among populations. Evolution.

[b77] Wilson GA, Rannala B (2003). Bayesian inference of recent migration rates using multilocus genotypes. Genetics.

[b78] Wilson AC, Cann RL, Carr SM, George M, Gyllensten UB, Helm-Bychowski KM (1985). Mitochondrial DNA and two perspectives on evolutionary genetics. Biol. J. Linn. Soc.

[b79] Yu H, Frommer M, Robson M, Meats A, Shearman D, Sved J (2001). Microsatellite analysis of the Queensland fruit fly Bactrocera tryoni (Diptera: Tephritidae) indicates spatial structuring: implications for population control. Bull. Entomol. Res.

[b80] Zayed A, Packer L, Grixti J, Ruz L, Owen R, Toro H (2005). Increased genetic differentiation in a specialist versus a generalist bee: implications for conservation. Conserv. Genet.

